# Presence of antibodies against tick-borne encephalitis virus in sheep in Tunisia, North Africa

**DOI:** 10.1186/s12917-020-02651-6

**Published:** 2020-11-12

**Authors:** Médiha Khamassi Khbou, Rihab Romdhane, Asma Amina Foughali, Limam Sassi, Vanessa Suin, Mourad Rekik, M’hammed Benzarti

**Affiliations:** 1 Laboratory of Infectious Animal Diseases, Zoonosis and Sanitary Regulation, Institution of Agricultural Research and Higher Education, Univ. Manouba, National School of Veterinary Medicine of Sidi Thabet, 2020 Sidi Thabet, Tunisia; 2 Laboratory of Parasitology, Institution of Agricultural Research and Higher Education, Univ. Manouba, National School of Veterinary Medicine of Sidi Thabet, 2020 Sidi Thabet, Tunisia; 3 Viral Diseases Service, Sciensano. Rue Juliette Wytsmanstraat 14, 1050 Brussels, Belgium; 4 International Center for Agricultural Research in the Dry Areas (ICARDA), P.O. Box 950764, 11195 Amman, Jordan

**Keywords:** Tick-borne encephalitis, Sheep, Antibodies, Seroneutralisation, Tunisia

## Abstract

**Background:**

Tick-borne encephalitis virus (TBEv) is a flavivirus that circulates in a complex cycle involving small mammals as amplifying hosts and ticks as vectors and reservoirs. The current study aimed to investigate the presence of TBEv in Tunisian sheep. A sample of 263 adult sheep were selected from 6 localities where *Ixodes ricinus* is well established. Sera were screened using ELISA for TBEv IgG detection, then the doubtful and positive sera were tested by the seroneutralisation test (SNT) and screened for West Nile Virus (WNv) IgG for cross-reaction assessment.

**Results:**

The ELISA for TBEv IgG detected one positive serum and 17 borderlines. The SNT showed one positive serum among the 18 tested, giving an overall antibody prevalence of 0.38% (95% CI = 0.07–2.12%). All but one serum tested negative to WNv ELISA. None of the sheep farmers reported neurological signs among sheep or humans in their households.

**Conclusions:**

The results may indicate the circulation of TBEv for the first time in Tunisia and in North Africa. Further studies based on either virus isolation or RNA detection, are needed to confirm the presence of TBEv in North Africa.

## Background

The Flavivirus genus comprises several arboviruses of both medical and veterinary importance, such as West Nile virus (WNv), Usutu virus (USUv), Yellow fever virus (YFv), Louping ill virus (LIv) and closely related subtypes (Turkish Sheep Encephalitis virus (TSEv), Greek Goat Encephalitis virus (GGEv) and Spanish Sheep Encephalitis virus (SSEv)), and Tick-borne encephalitis virus (TBEv) [1]. The latter is the causative agent of the most important zoonotic arboviral disease in Europe [2]. Three subtypes of TBEv were identified according to their geographic distribution, namely European (TBEv-Eu), Siberian (TBEv-S), and Far Eastern (TBEv-FE) [3].

 In the European Union countries, the number of human cases was estimated to 2000–3000 during the last decade, while in Russia it was about 1500–2000 cases per year [4]. According to several authors [5, 6], climate changes foster the expansion of the TBEv to western Europe and increase its occurrence to new areas. Indeed, in 2016, a marked increase of TBE cases in France was reported compared to the three previous years and the number of cases reached 29 [7], while the first human case was reported in The Netherlands [8].

The epidemiological pattern of TBEv is complex; it involves ticks and a wide variety of small mammals as their hosts [5]. TBEv occurrs in the so-called natural foci and their size can be from small to large even in regions where its main vector, is frequent [9], the reason for that patchy pattern is not completely understood [10]. *Ixodes* (*I.*) *ricinus* is the principal tick vector of TBEv in Europe and the prevalence of viral RNA in questing ticks, exceeds rarely 1% even in areas with high TBE human incidence [11].

It’s assumed that only one-third of human patients develop a biphasic course of illness [12]. After an incubation period of 7 to 10 days, the first stage is usually marked by myalgia, headaches, and fever reaching 39 °C that lasts up to 7 days. The second stage is characterized by meningitis, meningoencephalitis, meningoencephalomyelitis in 50, 40, and 10% of patients, respectively [13]. The lethality rate of TBEv-Eu ranges between 0.5 and 2% [14]. Although there is no treatment for TBE, a vaccine is available and recommended by the World Health Organization in highly endemic regions [15].

Tick-borne encephalitis was also reported in dogs [16] and horses [17], and both were found to develop a similar course of the disease as in humans. On the other hand, infected cattle, sheep, and goats seroconvert asymptomatically in most of the cases. In sheep, antibody response persists for 28 months after vaccination [9] and remains detectable using the Virus neutralisation test, at a low level, up to 4 and 6 years in sheep and goats, respectively [18] with a positive correlation between seroprevalence and age [19]. In European risk areas, seroprevalence in sheep ranges between 5.9% (213/3590) in Germany [20] and 15.02% (78/519) in Romania [21].

The main transmission routes for humans are primarily tick bites and to a lesser extent raw milk consumption from viraemic ruminants [22]. Indeed, consumption of unpasteurized goats’ milk was responsible for a TBE outbreak in 2010, in Hungary as reported by Balogh et al. [23].

For multiple reasons, serology is a useful tool for TBEv antibody detection: (i) it’s cheaper and easier than RNA detection (ii) it’s more reliable because of the patchy pattern of TBE occurrence and the low prevalence of TBEv even in risk area; (iii) it detects specific TBEv antibodies in grazing animals making them good sentinels, (iv) and facilitate TBEv detection in pre-screened areas. Both ELISA and seroneutralisation tests (SNT) are used as serological tools for the detection of TBEv antibodies. Single TBEv-positive serological result should be carefully interpreted in areas without a TBE history and confirmation by the SNT, considered as the gold standard, is always required [24]. Moreover, knowing the other flaviviruses circulating in the same area could help to explain cross-reactions, mainly with LIv infection, while TBE should be considered for presence of antibodies against WNv in animal sera in at risk area [9].

In Tunisia, *I. ricinus* was reported in exclusively limited areas in five districts namely Jendouba, Béja, Bizerte, Nabeul, and Zaghouan [25–29]. These regions are characterized by relatively high altitude, presence of deciduous woodland and coniferous forest, high humidity associated with annual rainfall varying between 500 and 800 mm. In Tunisia, *I. ricinus* was collected either from vegetation by flagging [25, 26, 30, 31], or from animals, such as cattle [28], lizards (*Psammodromus algirus*) [27], and more recently from small ruminants [32]. These ticks were infected by several zoonotic bacteria, namely, *Borrelia burgdorferi* s.s., *B. lusitaniae*, *B. garinii*, *Rickettsia monacensis* and *R. helvetica* [26, 29, 31]. But to our knowledge, no zoonotic virus was investigated in Tunisian *I. ricinus* ticks or from similar North African regions. However, mosquito-borne flaviviruses, namely WNv and USUv viruses, were reported in equines in South West Tunisia [33, 34].

In North Africa, sheep are facing several highly pathogenic endoparasites (ex. *Haemonchus contortus*, *Fasciola hepatica…*)[35] and ectoparasites (ex. ticks, mange…)[36]. When cumulated to bacterial (ex. *Mycobacterium avium* subsp. *paratuberculosis*) [37], viral infections (ex. Bluetongue virus)[38], and bad herd’s management, the whole sector development is deeply impeded.

Since sheep and goats are the best sentinels of TBE occurrence in risk areas [20, 39] and TBEv prevalence in ticks is very low, the present study aimed assessing TBEv seroprevalence in sheep in well-established *I. ricinus* areas of north and north-east Tunisia.

## Results

All the interviewed farmers did not report any history of neurological signs in their family members. None of the farmers and household members did consume raw milk from small ruminants.

A total number of 29 *Rhipicephalus sanguineus* s.l. ticks were collected from 22 sheep, giving 9.3% (22/236) as the prevalence of ticks’ infestation. All the infested sheep were from Takelsa (Nabeul district), Amdoun (Béja district) and El Jouf (Zaghouan district) localities.

Out of the 263 tested sera, 18 reacted by ELISA-TBE, a ten-year-old cross-bred ewe from the locality of El Jouf (Zaghouan district) was positive (0.38%, 95% CI: [0.07–2.12]) and 17 were borderline sera (6.46%, 95% CI: [3.4–9.4]]). In each locality, there was at least one serum that reacted by ELISA-TBE except for the site of Sedjnene (Table 1).

**Table 1:** Data and status of seropositive animals to TBE in ELISA with serum concentration ≥ 63 VIEU/ml

Locality (district)	Flocks’ N°	Breed	Age (years)	Sex	ELISA (VIEU/ml)	Status to TBE-ELISA	Status to TBE-SNT	Status to WN-ELISA
**Takelsa (Nabeul)**	1	B	7	Female	90	Borderline	Negative	Negative
	1	B	10	Female	82.5	Borderline	Negative	Negative
	1	CB	5	Female	102.5	Borderline	Negative	Negative
	1	B	5	Female	80	Borderline	Negative	Negative
	2	CB	5	Female	115	Borderline	Negative	Negative
	2	CB	2	Female	105	Borderline	Negative	Negative
	4	CB	5	Female	95	Borderline	Negative	Negative
	4	CB	5	Male	105	Borderline	Negative	Negative
**Aïn Draham (Jendouba)**	4	CB	3	Female	105	Borderline	Negative	Positive
**Amdoun (Béja)**	2	CB	5	Female	77.5	Borderline	Negative	Negative
	2	CB	4	Female	65	Borderline	Negative	Negative
	3	CB	3	Female	63	Borderline	Negative	Negative
**El Jouf (Zaghouan)**	1	CB	5	Female	67.5	Borderline	Negative	Negative
	2	CB	10	Female	175	Positive	Negative	Negative
	2	B	4	Female	75	Borderline	Negative	Negative
**Cap Negro (Béja)**	3	CB	3.5	Female	77.5	Borderline	Negative	Negative
	5	QFO	5	Female	102.5	Borderline	Negative	Negative
	5	QFO	6	Female	80	Borderline	Positive	Negative

*B* Barbarine breed, *QFO* Queue Fine de l’Ouest breed, *CB* Crossbreeds, *VIEU* Vienna Unit, *WN* West Nile, *TBE* Tick-borne encephalitis, *SNT* Seroneutralisation test

Out of the 18 (positive and borderline) tested sera by SNT, only a six-year-old ewe of the Queue Fine de l’Ouest breed, from Cap Negro (Béja district) was confirmed positive and showed a SNT-titer of 1/25 (Table 1). All these sera were also tested for ELISA-WN; only one from the locality of Ain Draham was positive (Table 1). In sum, sera of two different ewes, from two different localities displayed positive reactions to SNT-TBE and ELISA-WN; both showed previously borderline results in ELISA-TBE. The one, that was positive in ELISA-TBE, did react neither in SNT-TBE nor in ELISA-WN.

## Discussion

Despite the low estimated seroprevalence (0.38%), the detection of TBEv antibodies in adult sheep, in the well-established area of *I. ricinus* in Tunisia, might indicate that TBEv is present in North Africa.

The methodology we adopted (screening sheep sera by ELISA and confirmation by the gold standard technique, SNT) [9, 40] supports the result of the only one positive animal that is strongly suspected. 

The seropositive ewe was born in the herd and the only explanation is that it was infected by ticks during grazing in the forest. However, as antibodies last for several years (4–6 years) in small ruminants [18], we could not determine precisely the infection period. It would be very interesting to explore ticks in this herd vicinity to detect TBEv, as the farm is located in the middle of a deciduous forest favourable to *I. ricinus* survival. Moreover, the location of the farm is about 7 km far from the Mediterranean Sea and almost 200 km far from the lower point of Sardinia (Italy). Since the north of Tunisia is situated on the migratory flyways linking Europe to Africa, the introduction of TBEv in this area could be possibly by migratory birds importing infected ticks [41].

Such low seroprevalence in sheep was recorded even in European risk areas and was explained by the patchy pattern of TBEv foci [20]. In our study, this low seroprevalence could be explained by several factors: (1) contrarily to other regions, in North Africa, *I. ricinus* infests cattle more than sheep [42]; (2) nymph is the most important tick stage involved in TBEv transmission [43], infesting reptiles, birds and small mammals, more than large mammals [44]; (3) the number of questing *I. ricinus* collected from vegetation in well-established Tunisian areas is limited and by far lower than that collected in Europe (unpublished observations); (4) generally, the infection prevalence of TBEv in ticks never exceeds 1% [11]. Knowing other flaviviruses circulating in the country, could give more insights into the region about the occurrence of cross-reactions in serological tests, which is very seldom, only in some cases, such as to Louping ill virus [9]. In our study, it’s less likely that the only positive serum was due to cross-reactions. On one hand, the probability of Louping ill virus (LIv) occurrence in Tunisia is almost nil. In part because the LIv has never been reported from African countries and because the LI infection causes a severe clinical course in sheep [45–47], whereas none of our sampled animals showed neurological signs. On the other hand, the only positive serum to TBEv was negative to WNv by ELISA.

The choice of September as animal sampling period was based on the reported *I. ricinus* peak of activity. Indeed, the maximum activity of *I. ricinus* larvae and nymphs in Tunisia occurs between March and August, whereas *I. ricinus* adults’ activity occurs between February and April [27, 32]. We assumed that, if *I. ricinus* infesting sheep were infected, and considering the delay for IgG production, the exposed sheep would be seropositive during the sampling period (September). Although viraemia is very limited in time, TBEv antibodies in small ruminants were shown to last up to 6 years [18].

As only one seropositive animal was detected, risk factors such as sex, age, breed, and regions could not be assessed. Further investigations in the same regions should be undertaken to confirm the virus circulation and to study the epidemiology of TBE in Tunisia.

Sheep of our study were infested by *Rh. sanguineus*, which is not known to be vector of TBEv [16]. The absence of *I. ricinus* tick on the studied animals could be explained by the period of sampling that did not correspond to the adult *I*. *ricinus* activity in Tunisia [42]. In Europe, *I. ricinus* and *I. persulcatus* are the most important vectors of TBEv [48]. However, it was shown that *Rh. appendiculatus* co-feeding with TBEv-infected *I. ricinus*, efficiently transmit TBEv even if the host does not develop detectable viraemia [49]. Further investigations should be implemented in Tunisia to detect TBEv in *I. ricinus* and to check if other tick species could be involved as TBEv vectors.

The introduction of TBEv in Tunisia could also be possible by live sheep importation. Indeed, the last importation of live sheep from Europe to satisfy the demand’s increase for Aid El Idha (sheep slaughtering Muslim feast) occurred from Romania in 2012 [50]. Several imported sheep were kept by private sheep farmers (personal observations) and may have been mixed with local animals and this might have caused the autochthonous sheep infection by TBEv.

The introduction of TBEv could pose a problem mainly for the local population in northern Tunisia. From one side, the geographic distribution of *I*. *ricinus* is restricted to the North of the country where contact between ticks and humans, is most likely to occur for forest guards, animal keepers during grazing in the mountain, and ecological tourists. From the other side the consumption of unpasteurized raw sheep milk could expose humans to the TBEv, through the very appreciated Sicilian local cheese, made with unpasteurized milk produced by Sicilo-Sarde ewes [51]. This sheep breed is the only dairy sheep breed in Tunisia, reared only in the north of the country.

## Conclusions

The present finding reports the eventual TBE autochthonous infection for the first time in North Africa and triggers several questions about the possible introduction of TBEv in Tunisia. As the seropositive sheep was born in Tunisia, we can state that a possible new TBEv focus was detected for the first time in Tunisia.

The low seroprevalence in sheep is not enough to set off an alert for human health decision- makers, particularly with the absence of virus isolation and human cases. Further investigations are urgently needed to estimate the seroprevalence of TBEv in humans and animals. Ticks should be collected in the surrounding areas of the farm where the TBEv antibody-positive sheep was found. When isolated in Tunisia, the occurring TBEv subtype could be identified to determine TBEv reservoirs among Tunisian domestic and wild animals, and to estimate the infection prevalence of TBEv in *I. ricinus* ticks. Thus, TBE should be considered in every seropositive serum to WNv in at risk area to avoid prevalence overestimation due to false-positive sera. The understanding of TBE’s epidemiology would pave the way to set up adapted public health control measures.

## Methods

### Study area and sampled animals

**Fig. 1:**
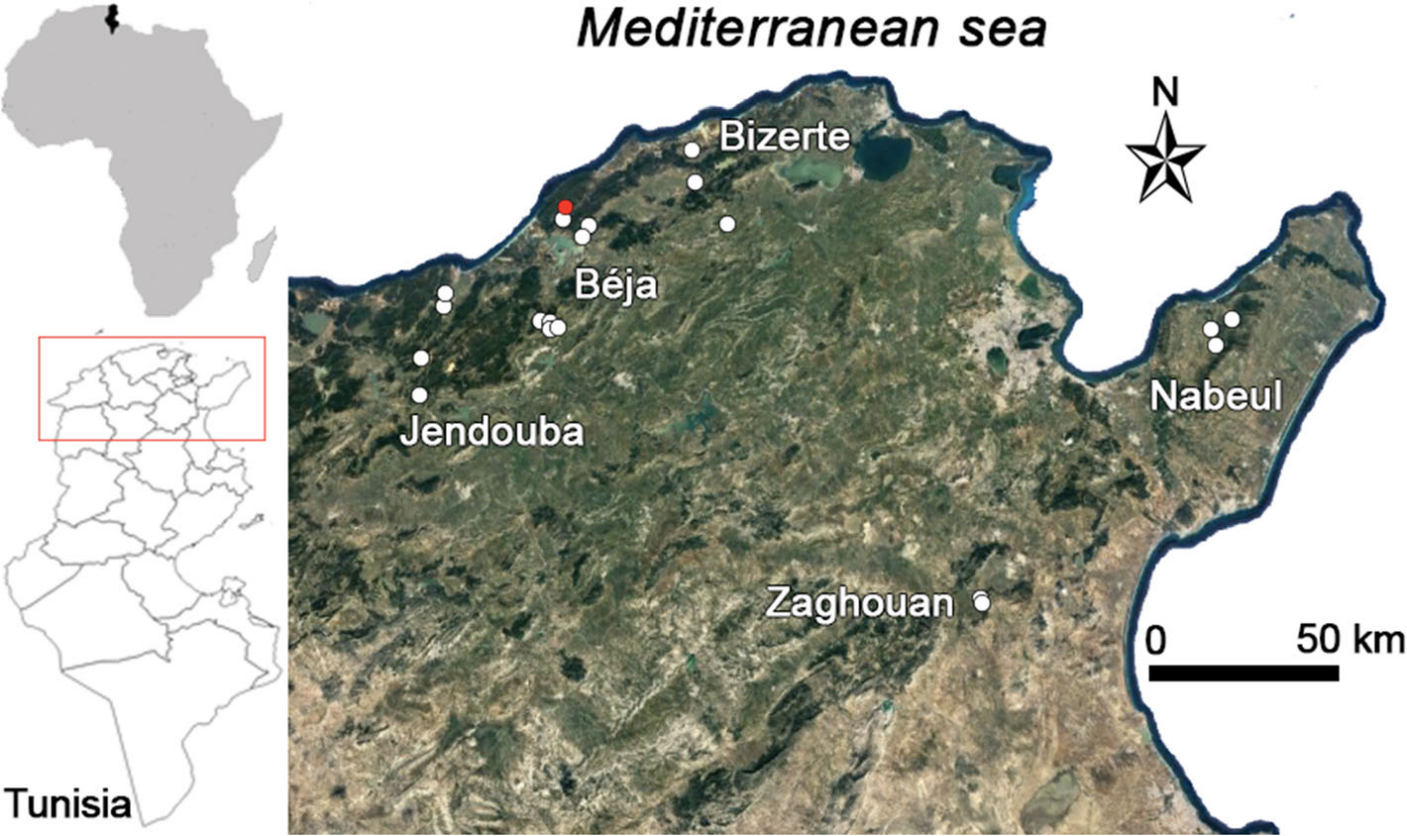
Map of Northern Tunisia indicating the sheep farms’ locations (white dots) with the names of the corresponding districts and the farm where the positive serum to tick-borne encephalitis virus was detected (red dot). The map was created in QGIS 3.12.2 [55]

A cross-sectional study was carried out during September 2019. A total number of 289 sheep from 22 small to middle-sized and extensively managed sheep farms were sampled. The sheep flocks are located in 6 localities in Northern Tunisia where *Ixodes ricinus* was reported [25–28] (Table 2; Fig. 1).

**Table 2:** Characteristics of surveyed sheep farms and their locations

Locality (District)	Flocks’ N°	Total number of sheep in sampled flocks	Seropositive by SNT/Total tested (%; 95% CI [lower - upper])	Latitude	Longitude	Altitude (m)
**Ain Draham (Jendouba)**	1	8	0/7	36°54'41"	8°44'56"	750
	2	9	0/7	36°53'38"	8°44'36"	750
	3	10	0/4	36°47'47"	8°40'46"	510
	4	20	0/9	36°42'52"	8°40'31"	570
***Subtotal***		*47*	*0/27*			
**Cap Negro (Béja)**	1	20	0/9	37°02’26”	9°06’26”	120
	2	7	0/2	37°01’56”	9°05’31”	120
	3	30	0/11	37°05’05”	9°03’18”	150
	4	25	0/14	37°05’05”	9°03’20”	120
	5	45	1/13	37°03’59”	9°02’44”	100
***Subtotal***		*127*	*1/49 (2.04; [0.36 - 10.69])*			
**Sedjnene (Bizerte)**	1	90	0/11	37°03’03”	9°27’39”	280
	2	120	0/12	37°11’33”	9°22’00”	30
	3	100	0/16	37°12’02”	9°25’07”	69
***Subtotal***		*310*	*0/39*			
**Amdoun (Béja)**	1	20	0/12	36°51’15”	9°00’25”	470
	2	30	0/20	36°51’14”	9°00’24”	470
	3	35	0/9	36°51’12”	9°00’23”	460
	4	20	0/10	36°51’21”	8°58’50”	530
***Subtotal***		*105*	*0/51*			
**El Jouf (Zaghouan)**	1	50	0/16	36°18’51”	10°06’03”	380
	2	50	0/23	36°18’55”	10°05’55”	390
***Subtotal***		*100*	*0/39*			
**Takelsa (Nabeul)**	1	50	0/20	36°50’23”	10°41’27”	110
	2	60	0/13	36°51’37”	10°44’20”	40
	3	10	0/5	36°51’36”	10°44’20”	50
	4	50	0/20	36°48’41”	10°41’47”	80
***Subtotal***		*160*	*0/58*			
**Total**	22	859	1/263 (0.38; [0.07 - 2.12])			

Italic values indicate subtotal for each district

*95% CI* 95% confidence interval, *SNT* Seroneutralisation test

All the sampled sheep graze in the mountainous forests throughout the year. To maximize the likelihood of antibodies detection probability, only sheep of at least 2 years old of both sexes were selected in all targeted flocks. In these flocks, all adult animals were sampled, excepting those of Sedjnene region, where 20 sheep were randomly sampled from each flock. All sheep were examined for the presence of ticks in the whole body, mainly the legs, ears, perineal and mammary regions. Sheep owners were interviewed about their health status and of their family members and were specifically asked if any neurological troubles were observed during the year anterior to sampling.

### Serological tests and tick identification

Only qualified persons (technicians and veterinarians) participated in blood collection. Five millilitres of blood was collected from the jugular vein of each sheep with a vacutainer in sterile dry tubes. After centrifugation at 3000 rpm (revolution per minute) during 15 minutes, sera were transferred to identified Eppendorf tubes and stored at -20 °C until used.

Sera were diluted at 1:50 and tested for the presence of anti-Tick-Borne Encephalitis virus antibodies using a commercial ELISA kit (IMMUNOZYM® FSME, IgG All Species, Progen, Heidelberg, Germany) coated with inactivated TBE virus. The ELISA plates were read with a spectrophotometer (Multiscan^TM^FC, ThermoFisher Scientific, Waltham, MA) at 450 nm wavelength to estimate the optical density (OD) of each serum. Positive controls (low level and high level) and five calibrators (human sera with known concentrations of anti-TBEv IgG concentrations) were included in the plates. As recommended by the manufacturer, the concentrations of tested sera samples measured in Vienna Units per millilitres (VIEU/ml) were estimated from a standard curve drawn from the OD of the calibrators. Serum was classified as positive when its concentration was ≥ 126 VIEU/ml, as borderline when its concentration was < 126 and ≥ 63 VIEU/ml, as negative when its concentration was < 63 VIEU/ml. To avoid over-interpretation due to cross-reactivity with other flaviviruses [9], sera that were either borderline or positive for TBEv were tested for anti-West Nile virus antibodies. For the detection of anti-WNv antibodies, a commercial ELISA kit (ID Screen, West Nile Competition Multi-species, IDvet, France) was used according to manufacturer recommendations. Briefly, sera were diluted at 1:2 and added to a plate coated with pr-E protein’s envelope of WNv. The optical density was read at 450 nm with a spectrophotometer and the ratio (S/N) of sample absorbance OD by mean negative controls’ OD was estimated for each tested serum. The serum was considered positive if S/N ≤ 0.4, doubtful if 0.4 < S/N ≤ 0.5 and negative if S/N > 0.5.

To minimize over-interpretation due to false-positive results among ELISA-positive and borderline sera, the gold standard Seroneutralisation test (SNT) was performed in the Viral Diseases Services of Sciensano Institute, Brussels - Belgium, as confirmatory test according to the protocol described by Roelandt et al. [52]. The test consisted of a rapid fluorescent focus inhibition seroneutralisation test (RFFIT-SNT) using TBEv Neudoerfl NCPV#848 as reference virus strain. Briefly, it consisted of diluting sera, including positive and negative controls at 1/9, 1/27, 1/81, 1/243 in 50 µL of Dulbecco Modified Eagle Medium (DMEM, Gibco, Netherlands), supplemented with 10% inactivated fetal calf serum. The positive control consisted of infected cells in contact with known TBEV positive human sera and the negative control consisted of infected cells in contact with a known TBEV negative human serum. Virus was added at a dose of 1.2 log 50% endpoint tissue culture infectious doses (TCID50%) to the diluted sera in each well, and incubated at 37 °C and 5% CO_2_ during 90 min and under the same conditions for 24 h after BHK-21 cells addition (35–45 × 10^3^ cells/100 µL per well). The fixation of plates was made with methanol at + 4 °C for 30 min. The detection of infected BHK-21 cells was performed by immunofluorescence staining, using two mouse monoclonal antibodies. After washing plates, the number of foci with infected cells was counted under the fluorescence microscope. The dilution of tested sera that neutralizes 50% (DIL_50_) of the virus served to estimate the seroneutralisation titer, according to the method of Reed and Muench [53]. A serum was considered positive if its titer was > 1/15 and negative when its titer was < 1/10. The titers between both values were considered doubtful. In the present study, only SNT positive sera were considered positive to TBEv.

Ticks collected from sampled animals were also preserved in identified tubes containing 70° ethanol. Ticks were identified using a stereomicroscope at species level according to the key of Walker et al. [54].

### Statistical analysis

The map showing farms’ locations was created in QGIS version 3.12.2 [55]. A satellite image layer was provided by the open source Google Earth database. The 95% confidence intervals (95% CI) for percentages and means were estimated [56].

## Data Availability

The datasets used and analysed during the current study are available from the corresponding author on reasonable request.

## Abbreviations

BHK: Baby hamster kidney
CI: Confidence interval
DIL50: Dilution
DMEM: Dulbecco Modified Eagle Medium
IgG: Immunoglobuline G
LIv: Louping ill virus
OD: Optical density
PBS: Phosphate-buffered saline
RFFIT-SNT: Rapid fluorescent focus inhibition seroneutralisation test
rpm: Rotation per minute
s.l: sensu lato
S/N: Optic density of the sample /optic density of the negative control
SNT: Seroneutralisation test
TBE: Tick-borne encephalitis
TBEv: Tick-borne encephalitis virus
TCID50%: Tissue culture infectious doses 50%
USUv: Usutu virus
VIEU: Vienna Unit
WNv: West Nile virus
YFv: Yellow fever virus
